# Study of Non-Newtonian biomagnetic blood flow in a stenosed bifurcated artery having elastic walls

**DOI:** 10.1038/s41598-021-03426-1

**Published:** 2021-12-13

**Authors:** Hasan Shahzad, Xinhua Wang, Ioannis Sarris, Kaleem Iqbal, Muhammad Bilal Hafeez, Marek Krawczuk

**Affiliations:** 1 Faculty of Materials and Manufacturing, College of Mechanical Engineering and Applied Electronics Technology, Beijing University of Technology, Beijing, China; 2 Department of Mechanical Engineering, University of West Attica, 250 Thivon & P. Ralli Str., Egaleo, 122 44 Athens, Greece; 3 Department of Mathematics, Quaid-i-Azam University, Islamabad, 15320 Pakistan; 4 Faculty of Mechanical Engineering and Ship Technology, Institute of Mechanics and Machine Design, Gdańsk University of Technology, Narutowicza 11/12, 80-233 Gdańsk, Poland

**Keywords:** Computational biophysics, Computational science, Computer science, Biophysics, Computational biology and bioinformatics, Gastroenterology, Mathematics and computing

## Abstract

Fluid structure interaction (FSI) gained attention of researchers and scientist due to its applications in science fields like biomedical engineering, mechanical engineering etc. One of the major application in FSI is to study elastic wall behavior of stenotic arteries. In this paper we discussed an incompressible Non-Newtonian blood flow analysis in an elastic bifurcated artery. A magnetic field is applied along $x$ direction. For coupling of the problem an Arbitrary Lagrangian–Eulerian formulation is used by two-way fluid structure interaction. To discretize the problem, we employed $P_{2} P_{1}$ finite element technique to approximate the velocity, displacement and pressure and then linearized system of equations is solved using Newton iteration method. Analysis is carried out for power law index, Reynolds number and Hartmann number. Hemodynamic effects on elastic walls, stenotic artery and bifurcated region are evaluated by using velocity profile, pressure and loads on the walls. Study shows there is significant increase in wall shear stresses with an increase in Power law index and Hartmann number. While as expected increase in Reynolds number decreases the wall shear stresses. Also load on the upper wall is calculated against Hartmann number for different values of power law index. Results show load increases as the Hartmann number and power law index increases. From hemodynamic point of view, the load on the walls is minimum for shear thinning case but when power law index increased i.e. for shear thickening case load on the walls increased.

## Introduction

In order to understand how hemodynamics factors effects the atherosclerotic disease FSI study is considered as powerful tool. FSI combines the wall stress with blood flow simulation study using computational fluid dynamics. Gao et al.^1^ studied the atherosclerotic plaque rupture which is associated with the stresses that act on or within the arterial wall. The tensile tresses on the wall is one of the primary triggers for vulnerable plaque rupture. They used high-resolution multi-spectral MRI (magnetic resonance imagining) to reconstruct carotid artery plaque morphology. They found degree of stenosis are related to shear stress distribution of wall. The tensile stresses of the wall are higher in luminal wall and lower at outer wall. Li et al.^2^ applied finite element analysis (FEM) to simulate stress within plaques of symptomatic and asymptomatic individuals. They found that plaques with higher stress are more prone to ruptured and symptomatic. Tang et al.^3^ studied the materiel propertied and plaque structure on stress distribution using 3D FSI model. They found increase in strain/stress is the cause of plaque rupture, and the study of region having plaque is helpful to understand rupture risk assessment.

The process of vascular wall injury resulted from the buildup removal of oily materials is known as Carotid atherosclerosis (CA). CA is the main reason of plaque and stenosis. Stenosis changes the blood flow which results to change in blood pressure and also cause the resistance in blood flow in the artery. The problems caused by stenotic are high shear stresses, high blood flow velocities, flow recirculation and compression of cyclic artery. Saloner et al.^4^ used unsteady and steady flows to examine the plaque in carotid bifurcation. They found that higher Reynolds number enhances the stress magnitude. Sharzehee et al.^5^ studied the 3D virtual stent with 90° curvature along with WSS and drug concentration. The numerical study was conducted for various values of Reynolds number. They found intensity of flow at 45° is stronger than 90°. Also increase in Reynolds number decreases the WSS. Huh et al.^6^ perform experiment to study the viscosity effects in stenotic vessels. Study shows recirculation zone for normal and abnormal blood analogues are 1.72 and 3.67 shorter than Newtonian analogue for Reynolds number less than 200. Also shear thinning effect are not significant for Reynolds number greater than 1000.

Blood flow studies is important to understand the factors behind cardiovascular disease development. In this regard, numerous experimental and theoretical studies have been carried out to understand non Newtonian blood effects in vessels of different sizes and geometries on their flow properties. Neofytou et al.^7^ studied non-Newtonian flow instabilities in a channel having sudden expansion. However, using whole blood of animals and human for experimental study was difficult task due to ethical issues and safety concerns. Additionally, for large blood vessels whole blood poses sever obstacle in PIV (particle image velocimetry) measurement. Ijaz et al.^8^ studied the pulsatile flow in stenotic region with permeable walls. Study shows that impact of stenotic magnitude can be reduced by introducing the nano particles. Ijaz and Nadeem^9^ presented the theoretical analysis of bio-nano-fluid through a curved stenotic channel. They used different nano particles to study the hemodynamics effects of stenotic region. The found that Au are more effect than Ag and Cu to reduce the hemodynamic. Ijaz and Nadeem^10^ theoretically investigated the heat transfer characteristics in a blood flow through atherosclerotic artery under the impact of nano fluid they found blood containing nano fluid mediation in blood is helpful to minimize the hemodynamic impact. A theoretical model of Casson hybrid nano fluid in a curved annulus was proposed by Shahzadi and Ijaz^11^. They observed that stress formation in a curve for non-Newtonian parameter is higher than that of viscus case. The mathematical model of viscus fluid flow between a concentric curved tubes is studied by McCash et al.^12^ Their study mainly related to application in endoscopy. The outcome shows that catheterization can be made more flexible by using a flexible peristaltic endoscope inside a curved sinusoidal tube^13^. Used Newtonian, Hybrid, and Casson model to study the flow characteristics in carotid bifurcation. They found Casson model results for axial velocity distribution are very different when compare to Newtonian fluid. While for blood flow simulation Newtonian model is ideal. Lopes et al.^14^ studied the carotid blood flow using FSI. They studied Two different viscosity models (Newtonian and Carreau). They found the stress on the wall is function of the viscosity and is greater for Carreau fluid case. Kumar et al.^15^ studied a three-dimensional (3D) FSI carotid artery. Hemodynamic parameters were analyzed to better understand the formation and evolution of atherosclerotic plaque in the carotid artery bifurcation by viewing physiological conditions first as normal and subsequently as hypertension disorder. They noticed that the geometry and flow characteristic of the carotid artery had a strong impact on hemodynamics.

Use of magnetohydrodynamis (MHD) decreases blood flow rate in the human artery system that are useful for the treatment of some cardiovascular diseases, hypertension and hemorrhage^16^. Tzirtzilakis^17^ developed the mathematical model for an MHD viscus blood flow. To obtained the flow features a numerical technique known as finite difference is applied. They showed the impact of magnetic field is greater for deoxygenated blood. Prakash et al.^18^ studied the magnetize blood flow in bifurcated artery having heat source. The study shows that there is increase in flow pattern and temperature of the blood. Haik et al.^19^ studied the biomagnetic fluid in the presence of thrombus. The results show there is strong change in flow behavior when the MHD increases, also increase in friction coefficient in noted. To look at the depth of blood flow mechanics with FSI and application of MHD on blood flow the readers are referred to^20–25^, and references therein.

Recently Anwar et al.^26^ conducted the analysis of biomagnetic blood flow in a bifurcated artery having elastic walls. Theoretical model of Newtonian fluid with applied magnetic field is considered. The study shows there is significant change in blood recirculation with the variation of Hartmann number and Reynolds number. Also magnetic field increases the WSS and pressure.

In this study we focused on the hemodynamics of the non-Newtonian magnetized blood flow flowing through stenotic artery. The walls of the artery are considered elastic. How Hartmann number, Reynolds number, shear thinning and thickening behavior effects the hemodynamics of the bifurcated region, elastic walls, and stenotic region were examined. The paper is arranged as follows in “Geometry of the problem” section the geometry construction is explained. In “Mathematical modeling” section the Power law fluid model is developed and non-dimensional form is obtained. In next session solution methodology is explained. In "Resultsanddiscussion" section results based on the outcome are discussed. In last section conclusion based on the outcomes is drawn.

## Geometry of the problem

**Figure 1:**
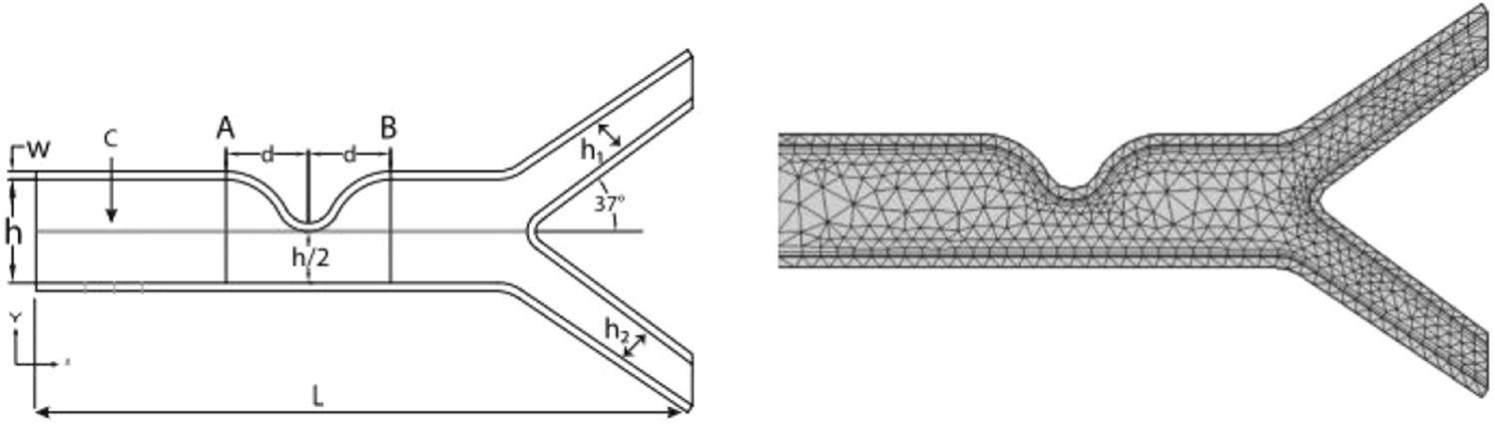
schematic diagram of the problem (left) and coarse mesh (right).

A typical geometric model having stenosis with bifurcation is assumed (see Fig. 1). The total length of the artery is $L = 6.34$, having diameter $h = 1$, which narrows down to 50% at the stenosis location. The elastic walls width is $w = 0.08,$ and the diameter of the other two artery is $h_{1} = 0.37 = h_{2}$. The angle at which the bifurcation artery is considered is 37°. The location of the line A is ($1.8,\,{\text{m}}$), where $0 \ll m \ll 1$, and $d = 0.8$ is the distance from the line A to the center of the stenosis. Note that lines A and B are to observe the velocity behavior before and after the stenosis. Further we assumed the walls are made up of isotropic and linearly elastic material having specific Poisson ratio and Young’s modulus. Which are defined as follows

1 $$\begin{gathered} v = \frac{\lambda }{{2\left( {\lambda + \mu } \right)}},\quad E = \frac{{\mu \left( {3\lambda + 2\mu } \right)}}{\lambda + \mu }, \hfill \\ \mu = \frac{E}{{2\left( {1 + v} \right)}},\quad \lambda = \frac{vE}{{\left( {1 + \mu } \right)\left( {1 - 2v} \right)}}, \hfill \\ \end{gathered}$$

where $\lambda$ = Lame coefficient, $\mu$ = Shear modulus, $E$ = Young’s Modulus, $v$ = Poisson ratio.where $E = 5 \times 10^{5}$ and value of $v = 0.49$^26^.

## Mathematical modeling

The two dimensional, incompressible non Newtonian power law biomagnetic fluid flowing through bifurcated artery is considered. We assumed low magnetic Reynolds number approximation, i.e. induced magnetic field is negligible compared to applied magnetic field^27^. The inlet flow is assumed parabolic while pressure at the exit is zero.

Basically Eulerian and Lagrangian description are used to study the continuum mechanics. The first one is suitable for fluid motion and later one is applied to study the solid mechanics. To describe the problems having mixture of solid and fluid domains, an ALE (arbitrary Langrangian Eulerian) is ideal^5^. The two dimensional governing equations for the fluid structure interaction can be written as^28^.

Continuity equation

2 $$\frac{{\partial \overline{u}}}{{\partial \overline{x}}} + \frac{{\partial \overline{v}}}{{\partial \overline{y}}} = 0,$$

Momentum equation

3 $$\rho \left( {\left( {\overline{u} - \overline{w}} \right) \frac{{\partial \overline{u}}}{{\partial \overline{x}}} + \left( {\overline{v} - \overline{w}} \right)\frac{{\partial \overline{u}}}{{\partial \overline{y}}}} \right) = - \frac{{\partial \overline{p}}}{{\partial \overline{x}}} + \eta^{*} \left( {\frac{{\partial^{2} \overline{u}}}{{\partial \overline{x}^{2} }} + \frac{{\partial^{2} \overline{u}}}{{\partial \overline{y}^{2} }}} \right) - \overline{\sigma } \left( {B_{0} } \right)^{2} \overline{u},$$

4 $$\rho \left( {\left( {\overline{u} - \overline{w}} \right)\frac{{\partial \overline{v}}}{{\partial \overline{x}}} + \left( {\overline{v} - \overline{w}} \right)\frac{{\partial \overline{v}}}{{\partial \overline{y}}}} \right) = - \frac{{\partial \overline{p}}}{{\partial \overline{y}}} + \eta^{*} \left( {\frac{{\partial^{2} \overline{v}}}{{\partial \overline{x}^{2} }} + \frac{{\partial^{2} \overline{v}}}{{\partial \overline{y}^{2} }}} \right)$$

where for power law fluid $\eta^{*} = K\dot{\gamma }^{n - 1}$. Where $\dot{\gamma } = \left( {2\left( {\frac{{\partial \overline{u}}}{{\partial \overline{x}}}} \right)^{2} + 2\left( {\frac{{\partial \overline{v}}}{{\partial \overline{y}}}} \right)^{2} + \left( {\frac{{\partial \overline{u}}}{{\partial \overline{y}}} + \frac{{\partial \overline{v}}}{{\partial \overline{x}}}} \right)^{2} } \right)^{{\frac{n - 1}{2}}}$.$\eta^{*}$ = stress tensor, $K$ = flow consistency index, $\dot{\gamma }$ = strain rate tensor, $n$ = flow behavior index.

The governing equation for solid displacement with no body forces is defined as

5 $$\nabla \sigma = 0.$$

Introducing the dimensionless variables, we have

$$x = \frac{{\overline{x}}}{h}, y = \frac{{\overline{y}}}{h}, \left( {u, v, w} \right) = \left( {\frac{{\overline{u}}}{{u_{0} }}, \frac{{\overline{v}}}{{u_{0} }},\frac{{\overline{w}}}{{u_{0} }}} \right), u_{s} = \frac{{\overline{u}_{s} }}{h}, p = \frac{{\overline{p}}}{{\rho u_{0}^{2} }}, \eta = \frac{{\eta^{*} h^{n - 1} }}{{K \left( {u_{0} } \right)^{n - 1} }},$$

6 $$Re = \frac{{\rho h^{n} u_{0}^{n - 2} }}{K}, Ha = \frac{{\overline{\sigma }B^{2} h^{2} }}{K} .$$

where $h$ = artery diameter, $u_{0}$ = inlet blood velocity, $Ha$ = Hartmann number, $Re$ = Reynolds Number.

With the help Eq. 6 of Eqs. (2–5) can be written as

7 $$\frac{\partial u}{{\partial x}} + \frac{\partial v}{{\partial y}} = 0,$$

8 $$\left( {u - w} \right) \frac{\partial u}{{\partial x}} + \left( {v - w} \right) \frac{\partial u}{{\partial y}} = - \frac{\partial p}{{\partial x}} + \frac{\eta }{Re}\left( {\frac{{\partial^{2} u}}{{\partial x^{2} }} + \frac{{\partial^{2} u}}{{\partial y^{2} }}} \right) - \frac{{Ha^{2} }}{Re}u,$$

9 $$\left( {u - w} \right)\frac{\partial v}{{\partial x}} + \left( {v - w} \right)\frac{\partial v}{{\partial y}} = - \frac{\partial p}{{\partial y}} + \frac{\eta }{Re}\left( {\frac{{\partial^{2} v}}{{\partial x^{2} }} + \frac{{\partial^{2} v}}{{\partial y^{2} }}} \right),$$

10 $$\nabla \sigma = 0.$$

where $\sigma$ is the strain tensor and for linear elastic walls it is defined as

11 $$\sigma = J^{ - 1} FSF^{T} ,$$

here $F = 1 + delu_{s}$ and $J$ is determine value of $F$. The relationship between second Piola–Kirchhoff stress tensor S and strain tensor $\varepsilon$ is defined as $S = C:\varepsilon$ and $C = C\left( {E,v} \right)$. Where strain tensor is defined as

12 $$\varepsilon = \frac{1}{2}\left( {\left( {\nabla u_{s} } \right)^{T} + \nabla u_{s} + \left( {\nabla u_{s} } \right).\nabla u_{s} } \right),$$

The parabolic inlet velocity is used and is defined as

13 $$u\left( {x,y} \right) = 3y\left( {1 - y} \right).$$

The pressure is considered zero at outlets further no slip is considered between fluid and solid.

## Solution methodology

To deal with FSI problem, the ALE method based on FEM has been employed to solve the system of partial differential Eqs. (8–10). Following the Galerkin finite element approach, the equations were converted into weak form and discretized^29^. More detail about ALE method can be found in Donea and Huerta^29^, Donea and Giuliani^30^, Kuhl et al.^31^, Mazumder^32^. To improve the accuracy of the solution, a hybrid mesh consisting of triangular and rectangular elements is generated. For velocity, pressure and elastic walls approximation an element pair P_2_–P_1_ is selected. The nonlinear algebraic system of equations is solved with the help of Newtonian’s method. While direct solver is applied to solve inner linear sub problems. The criteria for the convergence for nonlinear iteration is defined as

$$\left| {\frac{{\Im^{n - 1} - \Im^{n} }}{{\Im^{n + 1} }}} \right| < 10^{ - 6}$$

where $\Im$ is the general solution component. Figure 1(right) shows the coarser lever mesh grid of the problem. The whole domain of the problem is divided in to finite subdomain which is called element. The elements within the domain are approximated by $P_{1}$ and $P_{2}$ elements. A hybrid grid containing quadrilateral and triangular element is generated. In Table 1 WSS on the upper wall are calculated for different refinement levels (coarse to extremely fine) by keeping n = 1.3, Ha = 4 and Re = 200 fixed. One can see that there is decrease in absolute error with an increase in refinement level. The error at level six (extremely fine) is 0. Therefore, during the study, the extremely fine level is adopted.

**Table 1:** Grid convergence (for different refinement levels) at n = 1.3, Ha = 4 and Re = 200.

Refinement level	wall stresses on upper wall	Absolute error
1	0.290648	–
2	0.290281	0.000367
3	0.290309	0.000028
4	0.290596	0.000287
5	0.290622	0.000026
6	0.290622	0

## Results and discussion

A mathematical model of biomegnatic blood flow in a stenosis bifurcated artery is developed by considering linearly elastic walls. The system of nonlinear differential equations is converted into dimensionless form by using suitable scales. To discretized the problem, the ALE approach is applied along with suitable boundary conditions. An FEM approach is used to solve the system of equations. The numerical solution is obtained for different values of involved parameters. In order to get the physical insight of the problem the results are obtained by for different values of power law index n, Re and Ha. The graphical results are shown in velocity surface and contours form. Move rover line graphs for WSS and load are also plotted.

### Analysis of velocity profile inside the artery

**Figure 2:**
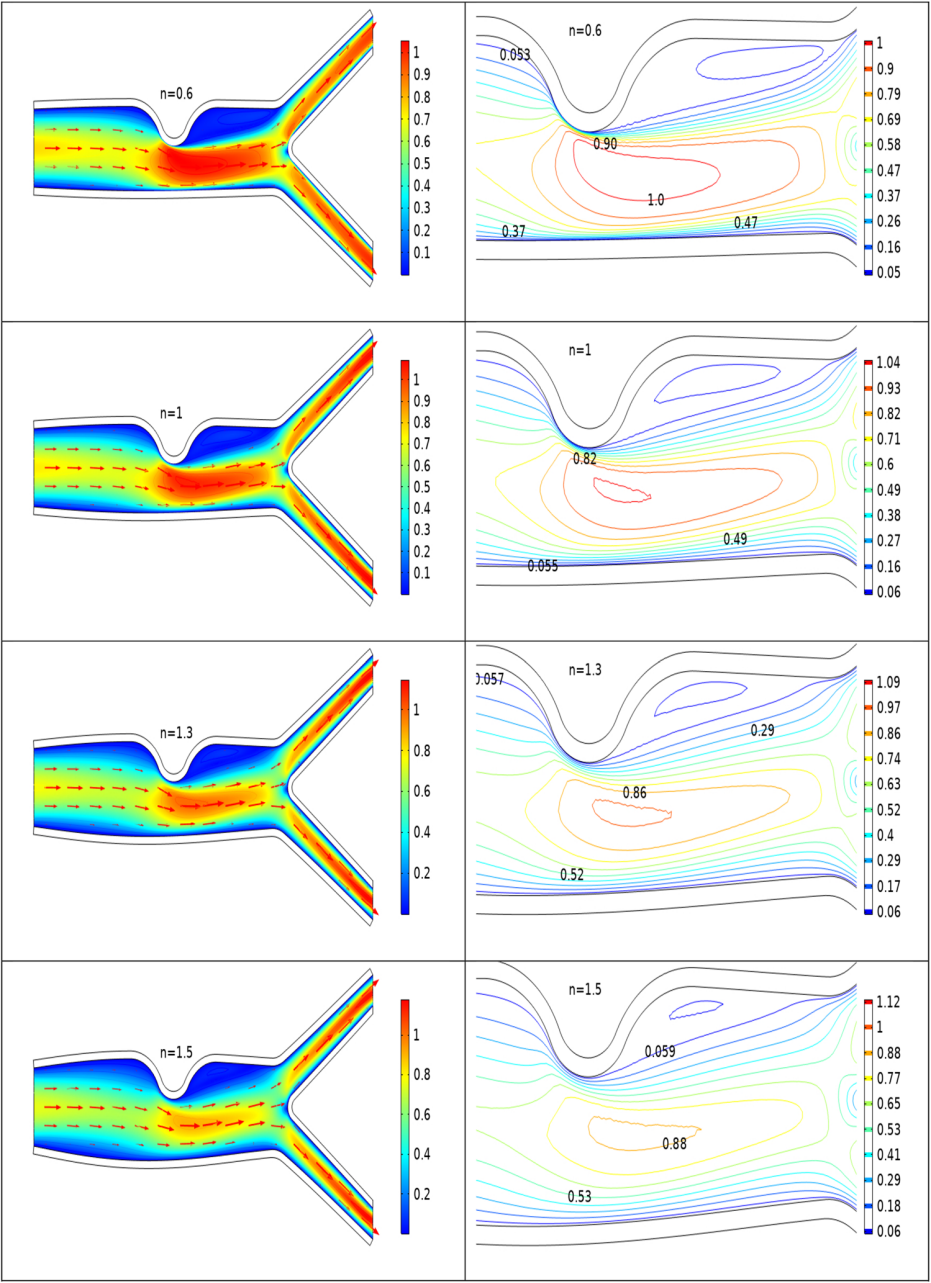
Velocity profile at Re 300 for variation of n.

**Figure 3:**
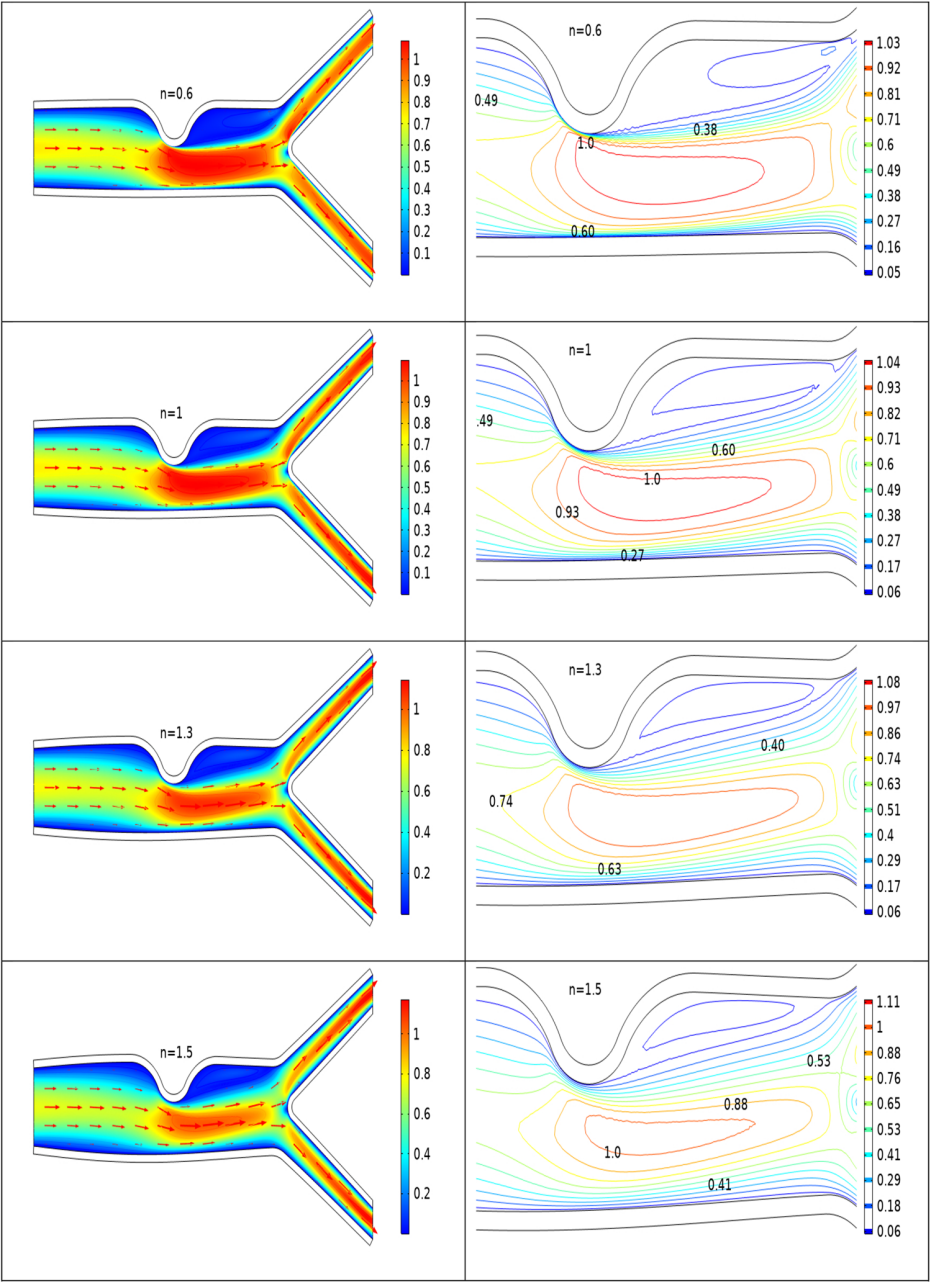
Velocity profile for various n at Re 500.

**Figure 4:**
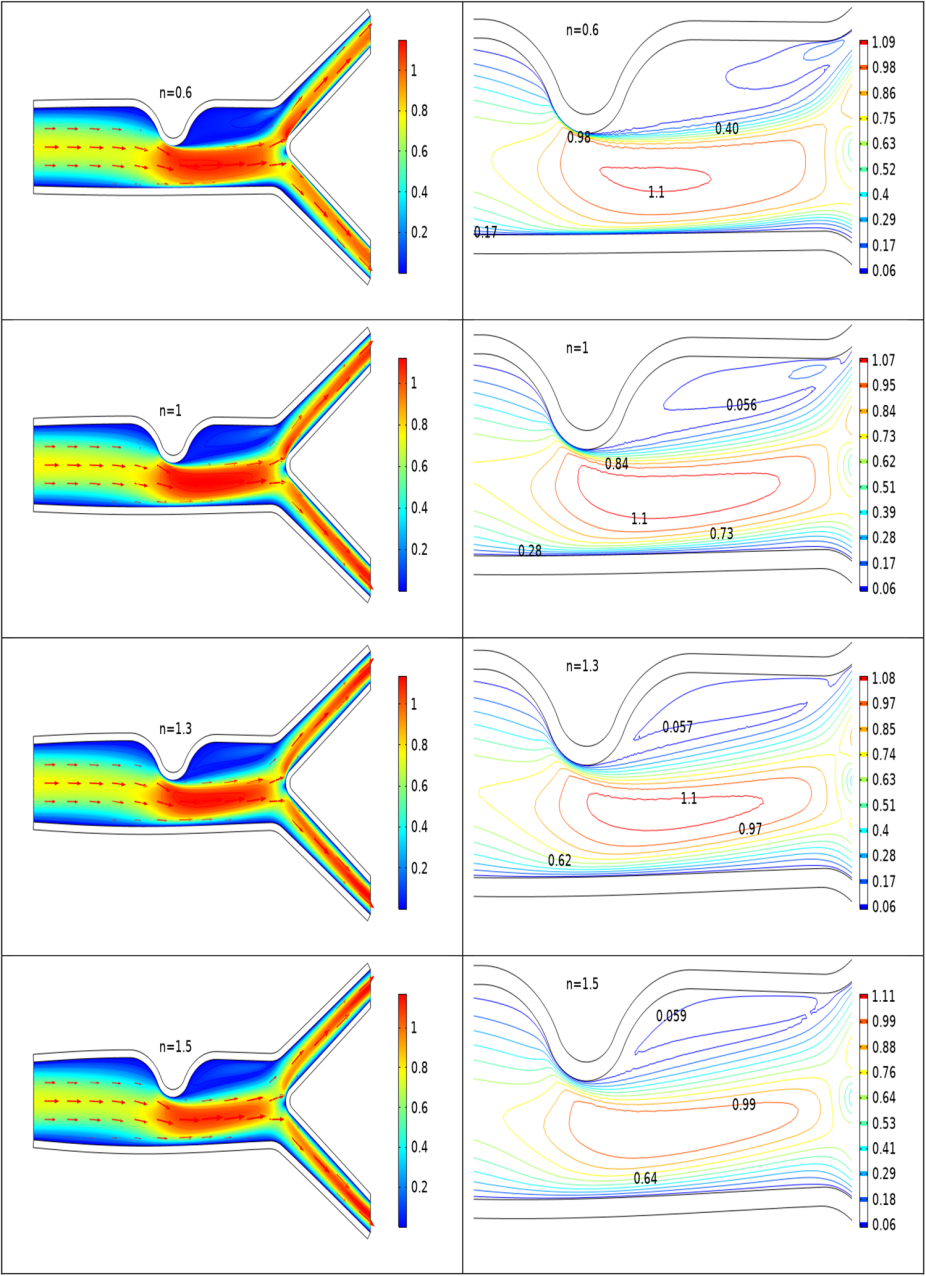
Velocity profile for various n at Re = 700.

**Figure 5:**
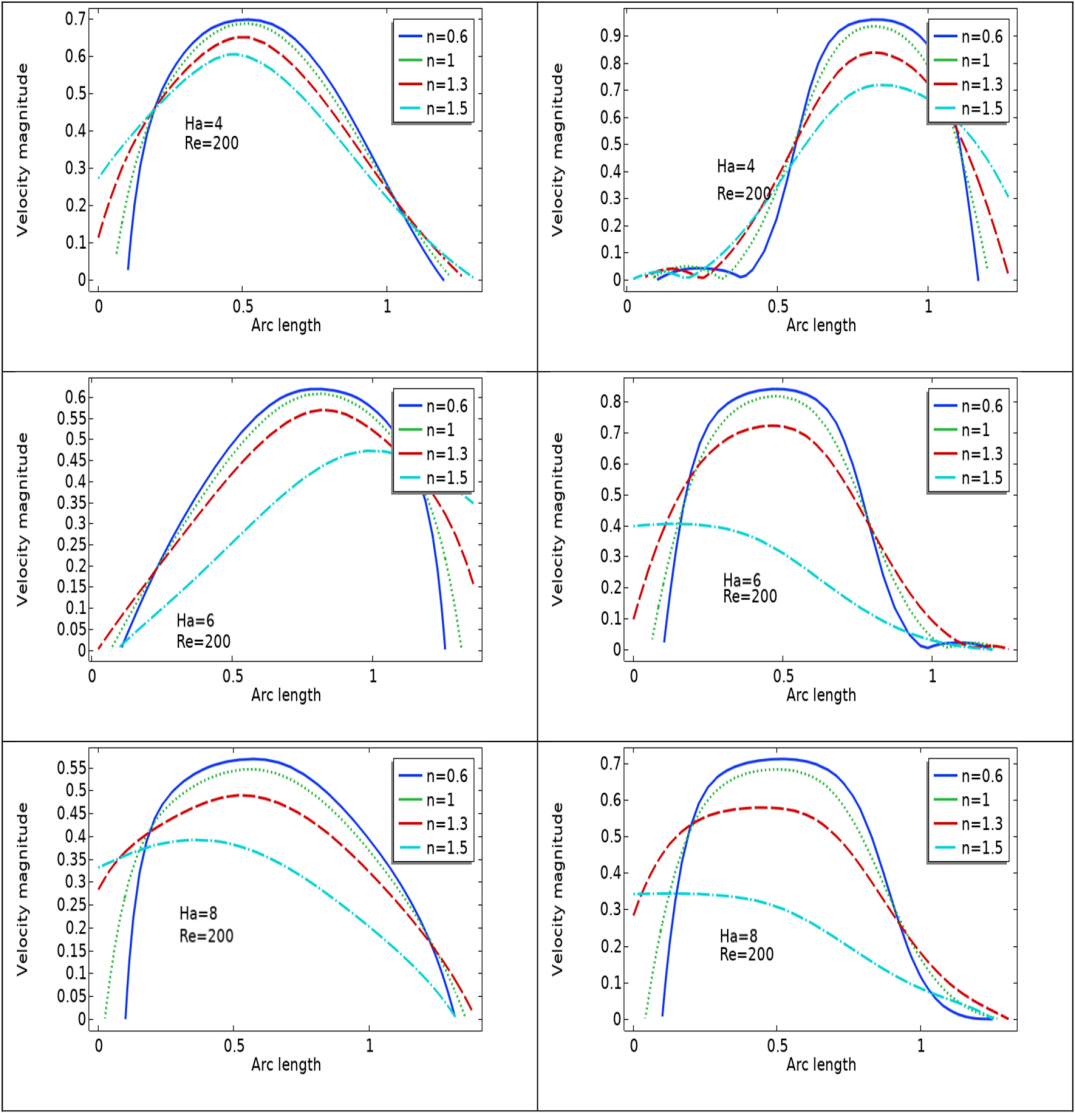
velocity profile for diffent Ha and n.

Figures 2, 3, 4 are plotted to see the impact of power law index between $0.6 \le n \le 1.5$ on velocity profile for Re = 300–700 by keeping Ha = 4 respectively. For increasing values of n the cavity area increases which clearly indicates when the value of n increases the pressure increases on the walls of the artery. Also one can see there is increase in recirculation area near the upper elastic wall. Physically this mean for shear thinning blood the velocity profile of the fluid is maximum and for shear thickening blood flow velocity decreases and exert pressure on the walls of the artery. Also for n = 1 the results are matched for the viscus case discussed by Anwar et al.^26^ Also near the stenosis velocity is maximum. In order to get an insight of the velocity behavior before and after the stenosis the findings between the points A and B is focused and are displayed in Figs. 2, 3, 4 (right). A direct relationship is observed between power law index and velocity profile. Also the recirculation pattern increases with increasing values of n. In Fig. 5 line graphs of the velocity (at location at A and B) for the variation of n and Ha is plotted. The left column shows the velocity before stenosis i.e. at the location A, while in right column the velocity magnitude at location B is displayed. The velocity magnitude decreases when the value of Ha increases. Also velocity magnitude has different behavior for the variation of n. Overall for both locations A and B velocity is decreasing function of n.

### Boundary loads and wall shear stresses

**Figure 6:**
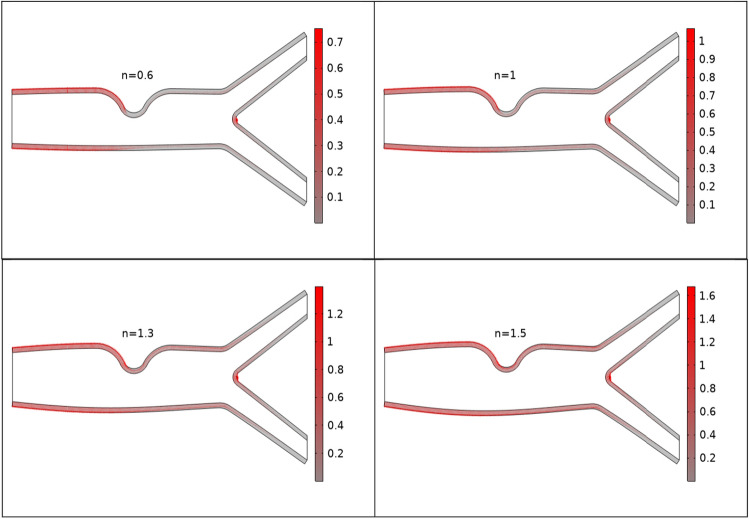
Boundary loads for the variation of n.

**Figure 7:**
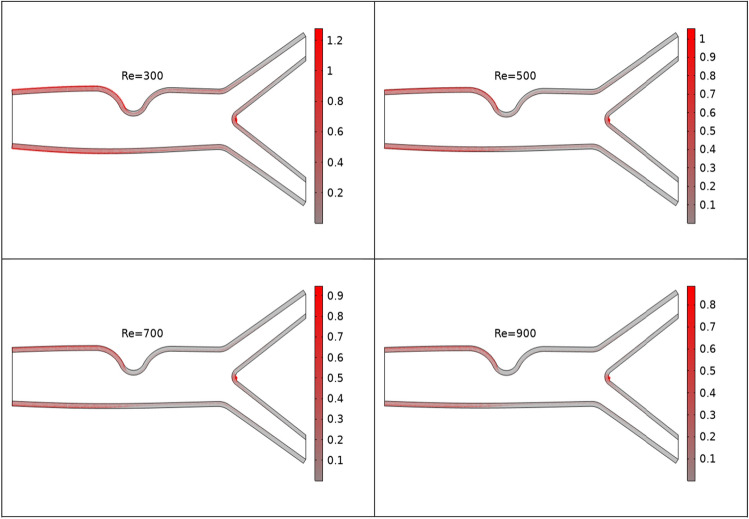
Boundary loads for the variation of Re.

**Figure 8:**
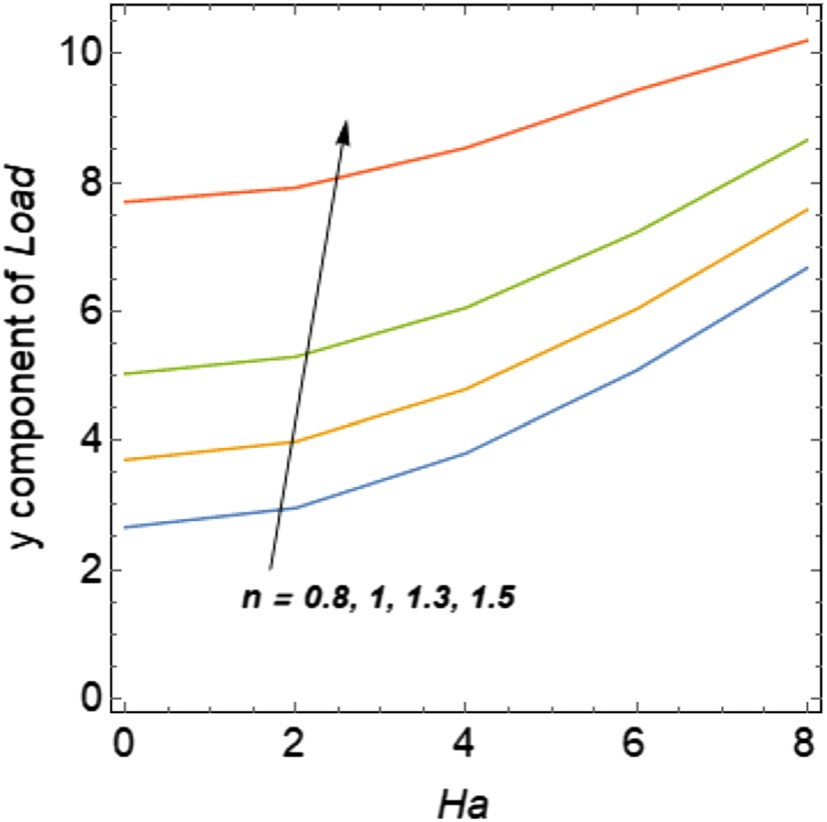
loads on the walls versus Ha.

In Figs. 6 and 7 boundary loads for different values of n and Re is depicted. Figure 6 shows loads on the walls of the artery is increases when the values of n increases. While Reynolds number has opposite effects on boundary load when compare to n see Fig. 7. In Fig. 8 y component of load at the upper wall is plotted against Ha for the variation of n. For increasing values of n load is increases.

Vascular pressure and wall shear stress (WSS) on the artery are two of the mechanical forces that are produced by blood flow through blood vessels. WSS are related to several developments and vascular processes such as vascular morphogenesis, angiogenesis, vascular tone and vascular remodeling. The WSS have been calculated for the variation of involved parameters and shown in Tables 2 and 3. For the variation of n and Ha the wall shear stress increases and consequently boundary load increases. The Reynolds number has opposite effect on the WSS when compare to n and Ha.

**Table 2:** WSS for variation of n and Re at Ha = 2.

n	Re = 200	Re = 300	Re = 400	Re = 500
0.6	0.101724	0.069024	0.052128	0.041891
0.8	0.141566	0.098395	0.075626	0.061547
1	0.192094	0.136533	0.106580	0.087774
1.3	0.286419	0.212295	0.169653	0.142043
1.5	0.347709	0.274095	0.223780	0.189704

**Table 3:** WSS for variation of n and Ha at Re = 200.

n	Ha = 0	Ha = 2	Ha = 4	Ha = 6	Ha = 8
0.6	0.099834	0.101723	0.107264	0.117453	0.129954
0.8	0.139735	0.141566	0.147703	0.159264	0.173330
1	0.190304	0.192094	0.198348	0.209791	0.222972
1.3	0.285158	0.286419	0.290618	0.296684	0.295043
1.5	0.348371	0.347709	0.344457	0.209528	0.227567

In Table 4 the numerical values of dynamic viscosities are calculated for variation of n and Reynolds number. Dynamic viscosity increases with and increasing values of n but Re has opposite effects on it.

**Table 4:** Dynamic viscosity for variation of n and Re.

n	Re = 200	Re = 300	Re = 400	Re = 500
0.6	0.024654	0.016562	0.012874	0.010655
0.8	0.029054	0.019175	0.014456	0.011698
1	0.035576	0.023722	0.017792	0.014235
1.3	0.049351	0.033972	0.025875	0.020830
1.5	0.059772	0.042898	0.033338	0.027210

## Conclusion

A mathematical model of the two-dimensional power law fluid passing through the stenotic bifurcated artery is constructed. The flow is assumed steady and incompressible. The shear thickening behavior of blood and its recirculation proximal to the stenosis is examined. Wall deformation with respect to WSS has also been analyzed. The governing equations are discretized using ALE technique. Finally resulting nonlinear system of equations are solved using Newton Raphson technique. The major findings of the outcomes are listed below.

- Stress magnitude increases for the shear thickening case.
- From hemodynamic point of view, the load on the walls is minimum for shear thinning case but when power law index increased i.e. for shear thickening case load on the walls increased.
- WSS increases for increasing values of power law index and Hartmann number.
- WSS decreases when Reynolds number increases.
- The dynamic viscosity of the blood increases with an increasing values of n which results to decrease in velocity magnitude.
- Load at the wall surface of the artery increases as the value of Ha increases.
- Boundary load is decreasing function of Re.

## List of symbols

$$\lambda$$: Lame coefficient
$$\mu$$: Shear modulus
$$E$$: Young’s Modulus
$$v$$: Poisson ratio
$$\eta^{*}$$: Stress tensor
$K$: Flow consistency index
$\dot{\gamma }$: Strain rate tensor
$n$: Flow behavior index
$$h$$: Artery diameter
$u_{0}$: Inlet blood velocity
$$M$$: Hartmann number
$$Re$$: Reynolds Number
$$p$$: Pressure
FEM: Finite element method
$L$: Length of an artery
$h$: Diameter of an artery
w: Width of elastic wall
$\rho$: Viscosity of the fluid
$\overline{u}$: $x$ Component of velocity
$\overline{v}$: $y$ Component of velocity
$\Im$: General solution component
$$\sigma$$: Strain tensor
$\overline{w}$: Mesh coordinate velocity
S: PiolaKirchoff stress tensor
$$\varepsilon$$: Strain tensor
WSS: Wall shear stresses
FSI: Fluid structure interaction
ALE: Arbitrary Lagrangian–Eulerian
